# Supplementary material for: Emergent order in epithelial sheets by interplay of cell divisions and cell fate regulation
Source: PLoS Comput Biol. 2024 Oct 14;20(10):e1012465. doi: 10.1371/journal.pcbi.1012465 (PMC11501039; doi:10.1371/journal.pcbi.1012465)
Supplement: Attachment — Submitted filename: Response.pdf [file pcbi.1012465.s002.pdf]

RESPONSE TO REVIEWERS

(Author responses in blue)

Reviewer's Responses to Questions:

Comments to the Authors:
Please note here if the review is uploaded as an attachment.

Reviewer #1: The article "Emergent order in epithelial sheets by interplay of cell divisions and cell fate regulation" looks at how an agent based model of cell fate choice may be used to better understand emergent order of cell-fate selection, and the effects cell division of fate selection.
This work begins with a brief motivation of the problem of cell-fate selection, and then proceeds to discuss some previous attempts in a brief manner. The authors outline their simple, yet very elegant agent based model which describes cell fate selection and division. To decide how cells transition between difference cell types (in turn, how their fate is chosen), the authors propose two different limiting probability distributions, and compare the models sensitivity to them, overserving how spatial patterning arises in both cases. To very different mechanisms in cell-fate selection are described: a cells fate is determined upon division, or cells may change their fate dynamically. A qualitative analysis is provided, comparing the behaviour from the different approaches.

I have a few comments and concerns regarding the articles fit for the journal PLoS Computational Biology.
1: The article requires a more concise literature review. Ising models have been well studied in physics with many people using them to describe cell behaviour in biological tissues. Some more effort to distinguish why this is a suitable approach to understand the emergent order of cell-fate selection, and also presenting these previous approaches would make this a much stronger article in its current form.

The Ising model is indeed the most well-studied model of statistical physics. I have now cited the original paper and a major text book reference, and further refer to its applications to describe cell behaviour in biological tissues.

Note that while the Ising model has been employed to study regulation of cell types in the case of reversibly switches, the interplay between cell division, different patterns of cell fate choice, and cell fate regulation, has not been studied yet and is novel in this paper. I am thus following a line of successful previous studies using lattice-based models to study cell dynamics and I therefore think that it is suitable, within natural limitations of such models. These limitations, and to which extent our model can predict realistic features, are now discussed in the Discussion section.

2: Upon reading the article, the work should be related back to the biological system in a clearer way. There are some rather interesting results that are observed in the article, but they are not related to a biological system.

I am now referring to those particular systems. In particular, the model applies to smooth epithelial sheets which are either flat or tubular, such as the basal layers of oesophagus and epidermis, and to some extent also to mammary gland epithelium.

3: Within the (rather nice and appealing) simulation results shown, they are presented for only single instance results. I would like to see some analysis perform with various simulations and a statistical analysis perform, to ensure confidence in the numerical results.

I apologise for not describing the simulation results correctly: In fact the plots showing simulated order parameters are not from single simulations but instead present the average of 80 instances of simulations with the same parameters. I have now made this clear in the text and figure captions, and error bars (standard error of mean) have been added.

4: I am concerned about this articles suitability for the journal PLoS Computational Biology. Typically, work requires a strong link to the biological system at hand. Relevant biological data needs to be incorporated. For example, if the article were to be considered for publication, I would suggest incorporating florescence imaging analysis to draw conclusion about which of the two proposed mechanisms best relate to the biology and how. Without the link, this work is much more of a theoretically valid approach.

This study does have a link to experimental research in that it proposes experiments to test the theoretical predictions. This article is not an experimental study, and experiments which could test the model predictions have not be done, so data is unfortunately not available. Please note that simple 3D fluorescent imaging data is not sufficient, since epithelial sheets are not entirely flat. The data first needs to be unfolded into a 2D arrangement, through topological computational algorithms which retain cell adjacency relationships.

While experiments/data analysis that would be required are feasible, they would require additional funding and staff to perform them, and many months or possibly years of study, which is outside the scope of this article.

In the revised version of the Discussion section, I now explicitly suggest experimental assays to test the model predictions. The work is thus highly relevant from an experimental perspective, because without providing the ideas for such experiments, they will never be done.

Due to its testable predictions, even without explicit tests via data (as not available), I believe this work is suitable for PLoS Computational Biology.

Reviewer #2: In this manuscript, the author develops an on-lattice model of two cell types (A and B) undergoing division and differentiation according to specific rules. The stochastic model evolves according to a Gillespie algorithm, with sites on the lattice randomly selected and evaluated for differentiation or division. The author introduces two order parameters (phi and tilde{phi}) and performs simulations to identify how changes in the cell-interaction strength and proportion of symmetric divisions impact pattern formation. The simulations produce random distributions of cells, clumps of cells, and nearly complete separation of different types of cells. The author also relates their model to Ising-style models from statistical physics and discuses phase transitions.

This is an interesting paper and an enjoyable read. I am not convinced that it is appropriate for PLOS Computational Biology, however, since the model is not tightly tied to biological data.

This study does have a link to experimental research in that it proposes experiments to test the theoretical predictions. This article is not an experimental study, and experiments which could test the model predictions have not be done, so data is unfortunately not available. Please note that simple 3D fluorescent imaging data is not sufficient, since epithelial sheets are not entirely flat. The data first needs to be unfolded into a 2D arrangement, through topological computational algorithms which retain cell adjacency relationships.

While experiments/data analysis that would be required are feasible, they would require additional funding and staff to perform them, and many months or possibly years of study, which is outside the scope of this article.

In the revised version of the Discussion section, I now explicitly suggest experimental assays to test the model predictions. The work is thus highly relevant from an experimental perspective, because without providing the ideas for such experiments, they will never be done.

Due to its testable predictions, even without explicit tests via data (as not available), I believe this work is suitable for PLoS Computational Biology.

I also have questions about the order parameters and variability across stochastic simulations, and I would suggest adding more references to the previous literature on voter models, order parameters, and cell-based models in general.

I am doing this now in the revised version, see my detailed answers below.

Main comments:
(1) Have the order parameters that the author defines been used in other studies, and are there other order parameters or quantitative approaches that could be used, such as pair correlation functions or pattern simplicity scores that might provide more information and further validate the observed differences?

Yes, the order parameters have been widely used, and are in fact the defining measures to quantify disorder-order phase transitions in complex systems. I do now reference the relevant literature.

Pair correlations, quantified through the correlation length, are indeed an alternative measure for phase transitions. It is known that the correlation length diverges at the phase transition. However, I do not think that studying those quantities offers additional information relevant for the article's conclusions, since the order parameter does already provide all information relevant for the scope of the study, namely, to show that it is a phase transition and for which parameters it occurs.

In Figures 1-3, would it be possible to include the mean order parameter values across 100 simulations with standard deviation bars? Can these order parameters capture differences in cluster size or pattern width?

I have done this now for n=80 repeated simulations (the shown curves were already the mean of 80 simulations, but I missed to mention this), and included error bars which represent the standard error of the mean. The order parameter does not capture the variance of cluster sizes. However, cluster sizes alone are not a good measure for identifying an interaction-driven

ordering phase transition, as the phenomenon of "percolation" may lead to the emergence of large, but fractal clusters, which are nonetheless completely random, and have nothing to do with order. The difference to genuine, non-random clusters is that clusters emerging from ordering are more bulky.

(2) At line 85 on page 5, I do not follow why phi = sum_i c_i is the same as phi = (N_A - N_B)/(N_A + N_B).

I have indeed forgotten the normalisation here, and corrected this. It should be phi = sum_i c_i/L^2, where L^2=N=N_A+N_B. The sum is sum_i c_i = sum_{c_i=+1} +1 + sum_i_{c_i =-1} (-1) = sum_{c_i=+1} 1 - sum_i_{c_i =-1} 1 = N_A – N_B.

Additionally, could the author add more intuition for what tilde{phi} is doing on an example pattern? Does tilde{phi} only capture checkerboard patterns with a pattern width of one grid square?

Yes, tilde{phi} only captures checkerboard patterns with a pattern width of one grid square. tilde{phi} is the same as phi taken from the staggered lattice tilde{c}, which is obtained from multiplying all sites with +/-1 in an alternating (checkerboard) pattern. That means that any patch with a checkerboard pattern in the original lattice will be a cluster of the same (staggered) cell type in the staggered lattice. Taking now the order parameter phi of the latter one gives a measure of macroscopic clusters in the staggered lattice, that is, macroscopic patches of checkerboard patterns in the original lattice. I have now amended the explanation of it to make that clearer.

Note that while this formulation of tilde{phi} only measures checkerboard patterns, it can be adjusted to other periodic patterns, by producing the staggered lattice by multiplying the lattice site values with a different periodic lattice function f(k,l), where k is the row index and l the column index (here: f(k,l) = (-1)^(k+l), but other definitions are possible).

(3) At line 8 on page 2, I suggest including biological images from some papers showing the different patterns that the author is referring to, or a cartoon illustration that shows different types of regular patterns and irregular domains.

I have now included an illustration of the different qualitative features of spatial cell type distributions to be found which reflect ordered structures. This is in a new figure (Figure 1).

Additionally, after Equation 3, could the author include references and more biological discussion about the assumption that stem cells maintain their position upon asymmetric division?

It is difficult to assess this in general, *in vivo*, as in reality both daughter cells are slightly displaced upon division. The assumption made in the model is rather that upon the delamination of a B-cell (to differentiate), a neighbouring cell divides, whereby the daughter closest to the previous position of the lost B-cell, is again a B-cell, so that the overall configuration does not change. In principle, this can only be assessed via intra-vital live imaging. And indeed, in the one study that is known to me to assess this, which is performed in mouse epidermis, it is shown that upon asymmetric division the neighbour configuration of cells does not change, that is, when a cell (B-cell) differentiates, a new cell that is inclined to

differentiate (also a B-cell) take their place [Rompolas et al. Science, 2016]. Furthermore, this can be assessed when asymmetric divisions have a slight inclination towards the basal membrane, as is the case in mouse oesophagus. In those cases it has also been found that the stem cell retains the position at the basal membrane, while the differentiating cell moves away from it [Frede et al, 2016].

I added those references in the "Model" section, and a few sentences in the Discussion section regarding this.

(4) For a general audience, it would be helpful to extend the discussion about how the model is similar to the voter model in the "Methods" section on page 5.

For omega = 0, J=0, the model is identical to the voter model. I have now added a brief description of the voter model and a reference to make this clearer.

(5) In the section "Simulation results", does c_i = 0 if it is empty, or is the number of cells always equal to the number of lattice sites?

There is no empty state ("vacancy"). Thus, yes, the number of cells is equal to the lattice sites: the loss of a B-cell is coupled to the immediate division of a neighbouring A-cell which immediately replaces the lost B-cell (by a new A- or B-cell depending on the type of division). One could also define a model in which empty sites exist and those "vacancies" travel through the B-cell population system until they reach a dividing A-cell. This would correspond to long-range crowding control mediated for example through mechanical forces or competition for diffusing growth factors. Such a model, which would correspond to different regulation mechanisms, would be equally worth studying. I did consider to study this, but decided against it for the following reasons:

1. Studying long range crowding control is not more or less important than juxtacrine regulation. Both occur in nature.
2. Including both model types in this work would require further comparison and exploration of wider parameter spaces. This would bloat the manuscript immensely and requires many more figures comparing the models.
3. Such a model has been shown in previous work (without contact inhibition nor cell type specific regulation) to produce qualitatively similar results as a model without vacancies [Greulich+Simons, PNAS 2016].
4. The absence of direct spatial coupling between neighbours could also be modelled as a mean field model, as known from statistical physics. While mean field models, for example as approximations for the Ising model or voter model, do not produce the quantitatively same results as the corresponding spatially coupled models, the qualitative results (e.g. existence of phase transitions) are usually recapitulated by them.
5. The model without vacancies is simpler and the theoretical mapping on systems where the phase transition structure has been understood is possible, in contrast to the model with vacancies.

Due to this reasoning I decided that studying only a model without vacancies is overall beneficial.

(6) In several figures, legends and information about the meaning of different colors is missing from the caption. For example, in Figure 1, what do black and white refer to? In Figure 2, what is the gray color in the patterns, and what parameter values do the colors of the curves in the upper two figures refer to? In Figure 3, what color curves represent what parameter values? Additionally, what are the initial conditions for the figures?

I am very sorry for this shortcoming. I have now added the relevant information.

I do not see any grey colour in Fig. 2, there are only black and white pixels.

I have now added the information about which colour corresponds to which parameter value in Fig. 2. This information was already given in Fig. 3 (In model 1, only J and L are parameters, which are given).

The initial condition is a random distribution of cell types, chosen with equal probability for each lattice site. I have added this now in the Methods section.

(7) In the Discussion section, I would suggest weakening the language surrounding conclusions and adding some discussion of model limitations, since the author is considering a simplified model with several assumptions (i.e., related to the model being a square lattice, having two cell types, etc).

Thank you for this comment. You are right that the limitations of this model need to be made clearer. I have now added a paragraph to outline what the model's limitations are. Furthermore, I made clear that the here discussed model can only provide suggestions to test qualitative features of spatial cell type arrangements (which I am confident it can), while only more detailed and realistic model can quantitatively test predictions on the data.

Minor comments:
(1) In Equations 5 and 6: what is J? I did not see this defined until later in the manuscript.

Now defined shortly after those equations (the equations are needed to define J).

(2) At line 60 on page 5: I think an "and" is missing in the list after "To summarize…"
(3) On page 6, typo: "fuzzy borders", not "fuzzy boarders".
(4) On page 10, typo: "this corresponds" rather than "his corresponds".

These are now corrected.

(5) On pages 10 and 11: I recommend moving more equations out of the paragraph form, so they appear on their own lines. This will make the paragraph more readable.

I have moved a few more referenceable equations out of the paragraph form. However, I left those equations which are shorter and represent only intermediate results in the paragraph form. I believe this makes it easier for the casual reader to see what are the important, referenceable equations.

(6) My understanding is that the author refers to "macroscopic domains" as patterns with large, separated regions of A and B cells. To me, "domain" means the domain of the simulation. I wonder if there is a better name for these types of patterns?

I am now using the term “patches”, as these are two-dimensional structures. However, the term “domain” for three dimensional structures is retained, as it is the most suitable term in this context and widely used as such.

(7) On page 8: The sentence “We now consider a particular class of spin systems,…” is too long and wordy and should be split into several sentences on page 8.

I have revised this sentence and have split it in two.

The sentence “The rate for this site to change..” is also long and difficult to parse on page 8.

I did not change this sentence, as I did not see how to do this without diminishing the information content. Please note that while technically this sentence is long, effectively it is already split in two by the colon, and the second part is a list, itself again split into two items.

Reviewer #3: The analysis presented in the paper appears mostly correct (subject to requested clarifications below) and is mathematically appealing. However, the biological conclusions presented are not substantiated, and a wide range of existing literature in this area is not discussed. My recommendation is thus that the paper is reconsidered after revisions, or rejected.

In the paper, the author introduces a cellular automaton model that investigates the effect of asymmetric vs symmetric cell divisions on patterning through lateral inhibition or lateral induction. He documents phase transitions in his model, i.e. he identifies critical signalling strengths for patterning to occur or not occur, as measured by a global order parameter. The author shows that the model is equivalent to an Ising model of spin interactions, and thus identifies parallels between the phase transitions in both models. These observations are interesting and well-suited for an audience of mathematicians or physicists.

In the model, the extent of patterning differs between scenarios considering symmetric or asymmetric cell divisions, and scenarios in which cell fate commitment is separate from the division. The premise of the paper is that such differences in patterning can hence be used to distinguish between different models of cell fate commitment and division based on imaging data alone.

I have the following concerns regarding the premise of the paper:

1)The model makes a few biological assumptions that are quite strong: The simulated tissue is under homeostasis, i.e. cell proliferation is assumed to be balanced by epithelial cell delamination. Is there a biological tissue that fulfils this assumption and which also patterns via lateral inhibition or lateral induction? Maybe there are plenty systems like this and I am ignorant of them, but no such system is cited in the paper.

Many homeostatic epithelial tissues like the adult oesophagus and epidermis are homeostatic and have cells signalling each other via the Notch signalling pathway which, in conjunction with Delta(-like) or Jagged, can lead to both lateral inhibition and induction (references now given in the text). The Wnt pathway which mediates a positive feedback via Wnt ligands via autocrine and paracrine routes can also lead to lateral induction. Thus, the relevant regulation mechanisms are present in homeostatic tissues. Which of these pathways are the most

relevant and whether the predicted patterning occurs is a matter that is still outstanding to be experimentally explored. There is evidence of clustering of stem cells in epidermis (e.g. [Jones et al., 1995]), but it is not clear whether this is due to Notch or Wnt signalling, or something else. No experiments with sufficiently processed image data (that is, 3D fluorescent images unfolded into 2D meshes), that would be suited to test corresponding quantitative models have yet been performed in tissues like epidermis and oesophagus to test this. The here presented work indeed suggests ways how to measure such patterns, to test different hypotheses on cell fate choice and regulation.

This is now been discussed in the Discussion section.

The biological references that the author is citing seem to be mostly systems in which cells divide without delaminating, or in which cells are not reported to divide. Some of the tissues are actively growing.

Please note that this is only part of the general induction, and the presence of those patterns in non-homeostatic, growing scenarios is to be seen as a motivation to explore whether they also exist in homeostatic tissues. I have now also added references to clustering in homeostatic tissue, namely, in human epidermis [Jones et al., 1995]

Similarly, the model makes the strong assumption that if a cell divides, a neighbouring cell delaminates, i.e. cell delamination and cell division are locally coupled. Is this a true phenomenon that can be seen in epithelia?

First of all, the model encompasses both cases (i) the A-cell divides and triggers a B-cell to delaminate, and (ii) the B-cell delaminates and triggeres the neighbouring A-cell to divide, not just case (i). It is in general difficult to assess this *in vivo*, as a direct measurements of this require intra-vital live imaging. Nonetheless, at least in mouse epidermis such a study has been performed [Rompolas et al., Science 2016], where it has indeed been shown that the loss (by delamination) of a differentiating cell is coupled to the immediate division of a neighbouring progenitor cell, which replaces the former in a way that the total configuration of cells does not change (Fig. 4 in [Rompolas et al., Science 2016]). This is very consistent with my model assumption of a lattice arrangement of cells. Furthermore, it has been shown indirectly, through mathematical modelling, for oesophagus in a Notch-inhibited mutant, that increased differentiation is coupled to increased division of immediate neighbours, although the causal order of events is not clear [Alcolea et al., 2014].

Hence, there is evidence, at least in the few tissues where it could be assessed, that delamination is coupled to the division of immediate neighbours.

Of course, long range coupling between delamination and division does play a role in many tissues and it would be equally relevant to study such a model (which would involve the presence of vacancies). Below, I will outline why I refrained from presenting such a model.

Even if we assume that the tissue is overall in homeostasis, wouldn’t we expect that the choice of delamination sites can affect the pattern just as much as the type of division events? If so, is it then justified that the model accounts for one of the effects but simplifies the other?

There are certainly regulation mechanisms in which delamination and division are not spatially coupled. In this case, aspects of the model would change (e.g. the inclusion of

empty, vacancy sites, which may in addition move through the lattice), and changes in the quantitative results would be expected, e.g. changes in the critical parameter values where a phase transition occurs. However, after careful consideration, I decided that it would not be beneficial to include such a model, for the following reasons:

1. Studying loss-division without spatial coupling is not more or less important than direct-contact regulation. Both occur in nature (see my response to the reviewers previous remark).
2. Including both model types in this work would require further comparison and exploration of wider (high-dimensional) parameter spaces. This would bloat the manuscript immensely and requires many more figures comparing the models. This treatment would become very long, complex and possibly confusing.
3. A model without spatial coupling of loss and division, which includes vacancies, has been shown in a previous work, in absence of cell-type specific regulation, to produce qualitatively similar results as a model without vacancies [Greulich+Simons, PNAS 2016]. It is thus expected that qualitative results would not change much in our case.
4. The absence of direct spatial coupling between neighbours is similar to a mean field model, as known from statistical physics. While mean field models, for example as approximations for the Ising model or voter model, do not produce the quantitatively same results as the corresponding spatially coupled models, the qualitative results (e.g. existence of phase transitions) are usually recapitulated by them.
5. The model without vacancies is simpler and the theoretical mapping on systems where the phase transition structure has been understood is possible, in contrast to the model with vacancies.

Due to this reasoning I decided that studying only a model without vacancies is overall beneficial.

I believe naming biological systems to which the model applies is essential for the premise of the paper. If no system exists to which the modelling assumptions apply, then the point that the model can be used to infer differentiation processes from imaging data does not hold. In that case there remains merit to the presented theory, but I believe that theory should then not be presented under a false premise.

As said above, epidermis and oesophagus (and possibly mammary gland), are the tissues where the model applies, at least in terms of the geometry and general existence of such signalling pathways. I am now mentioning this in the revised manuscript and added references. Nonetheless, whether the signalling is strong enough to have an effect on the spatial patterns is something that cannot be said without further experiments. The purpose of this paper is indeed to suggest experiments and to test these reasonable and testable hypotheses via modelling.

The Discussion section now also discusses in detail the limitations of the model, and possible experiments to test its predictions, or those of more detailed models.

2)Lateral inhibition is biologically typically mediated by interactions between the Delta and Notch signalling pathways. Much is known about these interactions. There are more than 2 decades of research on this topic, including a wide range of literature investigating how the coupling of Delta and Notch can lead to different patterns. For example, Wearing et al (2000) showed how different patterning spacings may be achieved through incorporating positive

feedback and Hadjivasiliou et al (2016) showed how long-range coupling can change the geometry of the pattern. Hawley et al (2022) investigated how patterns may change in time. – These are just a small selection of what's out there. Since there are multiple factors affecting the existence and shape of geometric patterns, how can we use the patterns as a way to identify different modes of cell differentiation?

I thank the reviewer for these references. I have now included those and discuss their relevance compared to the here presented work. I note that I had already included several references on Notch-Delta interactions, but the reviewer is correct that there has of course been much more research done on this. Yet, I believe that referencing all those works, which are probably dozens, would be beyond the scope of this work. I tried to follow a reasonable approach by presenting only a sample on those works.

Regarding the works which the reviewer mentions: Indeed, long-range coupling through Notch, via extended membrane protrusions, is a relevant mechanism, although not present everywhere. This can indeed change the periodicity of patterns, yet it does not change the qualitative feature that patterns have a short periodicity, on microscopic scales, in contrast to typical Turing-like patterns with periodicity on macroscopic scales, as produced by long-range morphogens. Similarly, dynamic switching of patterns in time [Hawley et al, 2022] would not negate the feature that those (short-length) periodic patterns exist. Furthermore, including such processes would involve an entirely different model, which may be equally relevant but not necessarily more than the presented one. As mentioned above, including additional model versions would require many more comparisons and figures and would go beyond the scope of a PLoS Comp article. After careful consideration, see the reasons above, I therefore decided to keep things simple and focus on nearest-neighbour interactions. Exploring different patterns under long-range interactions is a worthwhile endeavour, but would go beyond the scope of this work, in terms of length and complexity.

Finally, every single model used for inference of qualitative features, that is, hypothesis testing, can (and should) only cover a small range of possible hypotheses, and can generally only be used to reject those hypotheses covered by it. For a proper inference of qualitative features, one must test a multitude of model versions (and ideally reject all but one), some of them included in this work (nearest-neighbour juxtacrine signalling, and coupling of delamination and division), some not (long-range signalling, and no local coupling of delamination and division). A model which covers all possible hypotheses is often too complex to study and prone to overfitting. Hence I decided to keep it simple and study only a model covering a subset of possible hypotheses.

3)Is it true that we really don't know the difference between symmetric and asymmetric cell divisions in the biological contexts of lateral inhibition and lateral induction? One reference that the author cites, a nature paper by Sprinzak et al from 2010, uses live imaging to investigate how Notch signalling changes over time. In their pictures (e.g. figure 3D) it looks like Notch signalling gradually increases over a time period of multiple cell divisions, suggesting that cell fate and cell divisions are uncoupled (or coupled in a way that cannot be represented by the author's proposed model).

This work is done in *in vitro* cultures and not *in vivo*. The statements made in my manuscript can be applied to both *in vitro* and *in vivo* epithelial sheets, although the latter is indeed the regime where modelling provides most added insights, precisely because of the lack of direct observation (*intra-vital* live imaging is not always possible). Furthermore, the change in

Notch signalling over time, as reported in Sprinzak et al. 2010, is also associated to the transition of the cell culture from non-confluent to confluent, which likely affects cell-cell signalling. This is not something one would observe in homeostatic *in vivo* systems.

I have the following comments on the execution of the manuscript that I believe should also be addressed before acceptance.

4)The author refers to their simulation algorithm as a Gillespie algorithm while counting steps as 'Monte Carlo steps'. Is it a Gillespie or a Monte-Carlo simulation? The algorithm seems different from a Gillespie algorithm to me, since at each update step lattice sites are picked with equal probability. At the same time, the propensity of an event occurring is described to vary between lattice sites, since a cell can't divide if it doesn't have at least one differentiated neighbour. It is my understanding that in a Gillespie algorithm, a lattice site would be chosen with a probability proportional to the propensity of an event occurring at the site, not with equal probability for each lattice site.

It is indeed a slight variation ("version") of the classical Gillespie algorithm, a "random sequential update" scheme. The difference is that in addition to any event that changes the site configuration, "dummy events" are defined, which do not change the site configuration. The rates of those dummy events are chosen for each site at each time point, such that the sum of all rates is equal for all sites (i.e. for any site with maximal total rate, the dummy rate is zero, while for all those with lower total rate, the dummy rate is chosen to "fill up" the rates to be equal to the maximum rate). Then, all sites have equal total rate and can be chosen with equal probability, as done in the random sequential update. I have now clarified that this algorithm is a variation of the Gillespie algorithm, and given an additional reference where the scheme of introducing "dummy" rates is explained.

5)The author claims that the system reaches a steady state after L^2 lattice updates. Could this be demonstrated by showing the dynamics of the system over time, maybe in a supplementary figure? After L^2 lattice updates, each lattice site will on average have been updated once. My expectation would be that each lattice site would need to be updated multiple times before an equilibrium is reached.

I am very sorry for this misunderstanding. No, L^2 MCS means that each lattice site is on average updated L^2 times (that is, in total L^4 updates take place). Here I also corrected a typo, as the runtimes were L^2/2 MCS, yet this is still orders of magnitudes longer than as understood by the reviewer.

6) In figures 1, 2, and 3, one to five repeats of a parameter variation are shown. While it's nice to see the extent of stochastic variation by eye, I think it would be nicer to see means and error bars from a larger number of simulations. Especially in figure one, only one line is shown, so it's not evident from the figure whether this line is representative of the system behaviour.

I am sorry for not describing this correctly in the figure captions: each curve shown in Figs. 1,2,3 (now figs. 2,3,4) is not the result of a single simulation, but is the mean value of n=80 instances of simulations with the same parameters. In the revised manuscript, I am making this clear now and I also added error bars (standard error of mean) to the curves.

7)In figures 1, 2, and 3 it is difficult to see the numbers on the axes of the simulation images, and the axes are not labelled.

I have increased the tick label sizes to make them more visible. However, I do not understand what the reviewer means with “axes are not labelled”; in the version I have submitted, all axes are labelled, except for the lattice configurations. However, for the latter it becomes clear from the caption that each pixel denotes a lattice site; adding axes labels would require to significantly reduce the size of those figures and thus diminish visibility.

8)In figure 2, different colours correspond to different values of q, but it is not clarified which colour corresponds to which value of q.

I am sorry for not giving this crucial information in the figure caption. I have now added this.

9)The initial condition of the simulations does not seem to be mentioned. Does the outcome of the simulations depend on the initial condition?

The initial condition is a random distribution of spins/cell types, with each cell type having equal probability. This is now mentioned in the “Methods” section. The outcome does not depend on the initial condition in the disordered phase, but in the ordered phase, where large clusters emerge, at least the propensity to have clusters of either class dominating does so. However, the random choice of initial conditions mean that this is equally sampled, and as said above, the shown curves are in fact sample averages.

I have added a comment on the initial conditions.

These are cosmetic comments that the author may wish to consider, but that I believe are not necessary to be addressed:

10)There are two model versions used in the paper, ‘model 1’ and ‘model 2’. I believe the paper would be easier to read if the two model versions were named more descriptively. Maybe they could be ‘differentiation at division’ and ‘reversible differentiation’ (or ‘random’?)

I now call them model C, for “committed at division” and model R for “reversible”.

11)Some of the paragraphs are really long and could be visually and topically split up more to make the paper easier to read.

I have split paragraphs at a few places, in particular in the theory section, by placing equations in display mode.
