# Supplementary material for: Emergent order in epithelial sheets by interplay of cell divisions and cell fate regulation
Source: PLoS Comput Biol. 2024 Oct 14;20(10):e1012465. doi: 10.1371/journal.pcbi.1012465 (PMC11501039; doi:10.1371/journal.pcbi.1012465)
Supplement: Attachment — Submitted filename: Response_second_v2.pdf [file pcbi.1012465.s003.pdf]

RESPONSE TO REVIEWERS

Reviewer comments are in **blue**, my responses are in **black**.

Reviewer #1: The article "Emergent order in epithelial sheets by interplay of cell divisions and cell fate regulation" proposes a mechanism on cell fate regulation on epithelial sheets. The work uses an Ising model and couples cell fate and cell division.
The article is interesting, though not completely novel. Similar variants (and close relatives) of the Ising Model have previously considered similar interactions between cell fate and division.

While the Ising model has previously been used to address cell state/type switching in cell sheets, I am not aware of any work linking cell fate choice and its regulation with symmetric/asymmetric divisions through the Ising model. I note that the reviewer does not name such studies either. Hence, I am convinced that there is no other work that covers what the here-presented work does; it is therefore entirely novel.

Following my initial review, the authors have acknowledged the majority of my comments, except the work's relevance to the readers of PLoS Computational Biology, with the articles lack of data to validate, or even motivating the work. I do not accept that this would require significant funding as there is currently available data of sufficient quality within the literature.
I believe the work is of a high scientific quality, however, following on from my initial review, and the authors comments, I do not believe the article would be suitable for readers of PLoS Computational Biology. It is a heavily theoretical study, focusing mainly on analysing the theoretical model only. The authors do not relate their analysis to biological data, nor do they propose any real or relevant guidelines or directions for experimentalists to follow, driven by the work proposed here. I believe the work would be of interest to the scientific community, however, I believe it would appeal to those with a computational and theoretical understanding, in the work's current form. I would encourage the authors to pursue such avenues.

I added experimental images (Figure 1A-C) showing the different qualitative arrangements of cell types (disordered, alternating patterns, large-scale patches) as predicted by the model. This demonstrates qualitatively the features predicted by the model in experimental situations. In the discussion, I discuss which conclusions about cell fate choice can be made based on the qualitative features of one of those images.

Nonetheless, a quantitative test of the model on experimental data, as suggested by the reviewer, would be well beyond the scope of this manuscript. The reviewer claims that it would be possible to source image data of sufficient quality for a quantitative analysis from the literature; however, the reviewer may not have taken into account that since epithelial sheets are usually wavy and not entirely flat, too much information about the sheets' structure is lost when such a 3D situation is presented in 2D images. Thus, such images, which are commonly shown in papers, cannot be used. Furthermore, many images from in vivo epithelia show sections through the sheets which effectively only show one dimension of the epithelial sheet (see example now shown in Fig. 1C, which also clearly demonstrates the wavy structure of the epithelium). Despite a thorough search, I was not able to find images that contain sufficient information to reconstruct the epithelial sheet structure. I also note that the reviewer does not mention any examples of suitable sources.

In principle, it would be possible to get an appropriate representation of the data from the 3D raw image data of these experiments, by processing this raw data to identify cell-cell junctions and then computationally 'unfold' the wavy sheets into a flat configuration to compare with the model output. However, this is an advanced computational task and established software solutions to achieve this are not known to me. Thus, this would require the additional development and/or implementation of relevant algorithms and code from scratch. This is -- in addition to sourcing and processing the 3D raw data -- a substantial amount of work, usually requiring a dedicated researcher to engage with; thus additional funding would be required to employ staff on this project, in contrast to what the reviewer claims.

Please note that, as the reviewer says, this work is, and was from the outline, intended to be theoretical work, which yet makes robust suggestions for ways how to generate or process data to make the model predictions testable. The model has limitations with respect to the experimental situations, but these are clearly outlined in the discussion. Such work is within the remit of PLoS Computational Biology, as it is stated in the declaration of the scope of the journal that "Inclusion of experimental validation is not required for publication, but should be referenced where possible". Here, I am able to give such experimental references, that is, experimental images, which provide a qualitative experimental validation, but am not able to give a quantitative one.

Reviewer #2: The author has accounted for some of the referees' comments and improved the manuscript. It is an interesting study, and I have the following suggestions:

(1) My concerns about the proximity of this paper to biological data and predictions remain. The author mentions very general experimental tests in the discussion, but the model is a toy model and not tied to a specific biological system. Since several referees brought up the proximity to biology as a concern, I would suggest adding biological images as motivation for the different types of qualitative cell arrangements in Figure 1 and pointing out what cells would represent cell type A and B in these images. I think the cartoon illustrations of different types of patterns that the author added are useful, but it would help link the model to biology more tightly if each of these illustrations was accompanied with a biological picture of a system the model could be meant to describe. Many biological papers contain images that can be reprinted for free with permissions through the Copyright Clearance Center.

As suggested by the reviewer, I have now added such images in Figure 1, adding explanation of how this is linked to the model. In the discussion I discuss which conclusions about cell fate choice can be drawn based on the qualitative features in one of those images.

(2) References to biological literature need to be added in some places. For example, at ‘These dynamics mimic, for example, cells in the basal layers of epidermis, oesohagus, or organotypic cultures, which are smooth sheets or have tubular geometry, and which may be regulated through juxtacrine Notch-Delta or Notch-Jagged signalling.’ at line 252 on pg. 14 could have references to review articles for these observations.

I have now added the relevant references here.

(3) I would suggest rewriting the sentence “The order parameter is the defining measure for phase transitions in complex systems, and is thus the gold standard to identify them.” The

latter part (gold standard) is a strong statement and an opinion, so this should be clear. Pair correlation functions, order parameters, topological data analysis, radial distribution functions,… there are many choices for measuring the behavior of complex systems. I suggest adding a discussion with references to alternative quantitative approaches and a discussion of what they would add for future work (for example, the author mentions that the order parameter does not capture the variance of cluster sizes in their response, and this appears to be a feature of the simulations in Figure 3 that other quantitative approaches could capture).

I removed the qualifier "gold standard". It now just says that this measure is widely used to identify phase transitions in complex systems.

In the Discussion, I now point towards other measures and mention that they could discern further features. However, I don't believe that a comprehensive discussion of measures for phase transitions would be suitable at this point. It would rather be a distraction from the main thread of this manuscript.

Reviewer #3: The author has convincingly addressed my main concerns. In addition, the premise is more clearly stated, and the strengths and weaknesses of the paper are more openly discussed.

One of my main comments was a request to name possible biological systems to which the model could be applied. I asked about this since the author claims that the model suggests biological experiments. In the rebuttal, author mentions a reference (Jones et al 1995) which I thought was quite a nice way to address my comment. However, that reference does not appear to be cited in the revised manuscript. Surely, if the reference solidifies the premise of the paper, the readers should know about it?

I am sorry, this reference must have been lost in the last iteration of the manuscript. I have now added this reference in the beginning of the introduction and refer to it in the caption of Figure 1 (being related to panel 1C, which itself is taken from (Jones, Clinical Sciences, 1996)).
