# Supplementary material for: Emergent order in epithelial sheets by interplay of cell divisions and cell fate regulation
Source: PLoS Comput Biol. 2024 Oct 14;20(10):e1012465. doi: 10.1371/journal.pcbi.1012465 (PMC11501039; doi:10.1371/journal.pcbi.1012465)
Supplement: Attachment — Submitted filename: Response_third.pdf [file pcbi.1012465.s004.pdf]

RESPONSE TO REVIEWERS

Reviewer comments are in **blue**, my responses are in **black**.

Reviewer #1: The authors have accounted for my comments and I am happy to recommend acceptance. One small note: regarding "Persistent homology" in the parenthetical statement in the discussion, persistent should not be capitalized here.

I thank the reviewer for their recommendation for acceptance of the manuscript. I have corrected the typo.

Reviewer #4: The paper, 'Emergent order in epithelial sheets by interplay of cell divisions and cell fate regulation' uses a cellular automata model and theoretical insights from statistical physics to probe how the 'timing' of cell fate decisions, particularly with intercellular interactions, influences tissue structure. Overall, the paper is interesting and provides new insights into cell fate. However, I do echo previous reviewers' concerns around the fit for PLoS Computational Biology, as the manuscript is highly theoretical. (With apologies to the author for a) a late review that b) asks for even more work!)

I thank the reviewer for their generally positive assessment of the manuscript. I acknowledge that this is a theoretical paper, but – as argued in previous reviewing rounds – I believe that it is suited for PLoS Computational Biology, as it provides guidance for experimental approaches.

Putting those concerns aside, my main comments are around clarity of the manuscript and increasing the biological relevance:
- The author investigates cell patterning with two cell types. Does the author have any insight into how the system would differ with more than two cell types (two spin states would no longer be sufficient to describe the system, for example), including where this may appear in biology? Could this be added to the discussion section?

First of all, I admit that the term ‘cell type’, as used in the manuscript, can be ambiguous in this context. It could make more sense to classify A-cells and B-cells as two cell *categories*, a self-renewing cell category and a licensed/committed category (depending on the model version, ‘C’ or ‘R’), which are defined functionally by their lineage and proliferative potential, rather than by molecular markers, morphology, or detailed phenotype, as one would use to characterise cell types. Each category, A and B, may comprise multiple cell types, hence, the model can straightforwardly be generalised to multiple cell types. In general, the scenario considered in this manuscript is a lineage hierarchy from a stem cell type towards a differentiating type which leaves the epithelial sheet (‘delamination’), as is common in many epithelial tissues. In this hierarchy, we only distinguish cell types that are self-renewing but cannot delaminate, as ‘A’ cells, and those that can delaminate, ‘B’ cells. Within this scenario, multiple cell types can be taken into account by the model, and results would not be qualitatively different to the scenario we describe. However, the situation would be different with multipotent lineages or when self-renewing cells are able to delaminate, in which case the model does not apply.

I have now rewritten the relevant paragraph in the model section (where I believe it suits better than in the Discussion) to explain this.

- Is there any way to succinctly describe the method for how the neighbouring site j is chosen when a division event occurs? (This would be preferable to having to look up a referenced paper.)

The neighbours j are chosen randomly, with equal probability. I have added this statement now.

- Am I correct in saying the in model C only p^\lambda varies according to equations (5) and (6) and in model R only p^\omega varies according to equations (5) and (6)? If so, could this please be made clearer in the text?

Essentially this is correct. Eq. (8) indeed states that p^\lambda is fixed, which implies that only p^\omega varies in model R. In model C, in principle, both can vary, but since omega=0, variation in p^\omega is irrelevant. I have added a comment on this after Eq. (8).

- Page 5, final paragraph: in ignoring the superscript for the probability functions, is the implication that the cell division and cell fate change events behave the same way for the different neighbouring cell configurations? Could this be either corrected or made clearer in the text?

Since, as stated by the reviewer before, in model C only p^\lambda varies and in model R only p^\omega, it becomes clear from the choice of the model which, p^\lambda or p^\omega, is meant when neglecting the superscripts. As this is unambiguous, I neglect the superscripts for notational simplicity. I comment on this now after Eq. (8). Hence, in each instance, only one of the two, p^\lambda or p^\omega , has a functional according to one of the functions (5) or (6), not both at the same time.

- Page 7, line 137: The sentence starting with ‘For parameter’ is unclear - should this be ‘For the parameter J’? It should be made clear in the text that Fig 2 is for model C specifically.

I am sorry for this typo. This should mean ‘For model C’. This should also clarify the second comment.

- Sorry to be a pain, but are means and standard errors the most appropriate measures? Are the means and medians similar in your simulations, are the quantiles appropriately captured by the standard error? For example, in Fig 2. for the J=0.6, logistic example configuration, the configuration consists entirely of B cells, but this is not reflected in the top row plot of order parameter against signal strength. Apologies again for asking for 'more', but plots with medians and min/max or quantiles might better represent the sample simulations.

I gave it some thought, yet I am convinced that mean and standard error of the mean (s.e.m.) are the most appropriate measures to display, for the following reasons:

- The goal of this study are not quantitatively accurate predictions, but qualitative predictions, namely that phase transitions occur, upon parameter variation, at non-

zero parameter values. Since phase transitions are defined trough the **mean** order parameter -- more accurately, by the presence of an abrupt increase upon parameter variation for infinitely large systems -- it is essential to assess the mean and the slope of the mean as function of the parameters, which thus should be the displayed quantity.

- To demonstrate a phase transition, it needs to be shown that the slope increase with system size. However, typical visualisations of sample distributions that display median and quantiles, e.g. boxplots or violin plots, obscure the central trend, and therefore obscure the visibility of the slope, rendering this assessment difficult.
- As our goal is to show the behaviour of very (effectively infinitely) large system, ideally, we would simulate much larger systems, but we are limited through computational constraints. In very large systems, fluctuations vanish and thus predictions for such system should not include substantial sample variations. To overcome the shortcoming of 'finiteness' of our simulated systems, we assess a scaling of system sizes that shows the trend towards large system. However, any sample variation in our model is simply an artefact of the computational constraints, the small system size, and does not display real world variations. It would therefore be misleading to display sample variations. Display of the s.e.m., however, is required to estimate the error of our mean prediction.
- I do not quantitatively compare the simulations with experimental data, and do not assess significance or effect sizes. Hence, sample variations and confidence intervals are not required in that context.

- For Fig 3, it might be worth noting the different J scales for the different models in the caption.

This is now being noted in the caption.

A previous reviewer mentioned the 'grey' colouring in this figure (logistic, J = -0.3, q =0, Hill, J = -3.0, q = 0, J = -1.5, q = 0, for example). To assist readers, it may be worth noting that due to the resolution/magnification the 'checkerboard' patterning cannot be seen, or perhaps a zoomed in inset would be helpful?

I do note this now in the caption. I do not see a reasonable way to present a zoomed inset in a way that it would improve the visibility, since the figure is already very full.

- Page 9, top paragraph, would be good to have a summary (with biological interpretation) at the end, like the paragraph on Fig 2.

I added some sentences on the biological interpretation here.

Similarly, the second paragraph could use more biological insight. For example, for the parameter values in rows three and four of Fig 4., one cell type never dominates.

Here, a summary with biological insights is already given at the end of that paragraph. Nonetheless, I added a comment on the observation that a single cell type does not completely dominate any patch/pattern (Fig. 4) to the previous paragraph, where I believe it suits better.

The theoretical insights section would also be bolstered by biological interpretation.

I am not sure what kind of biological interpretation the reviewer expects here. The theoretical insights section is an analytical confirmation that the numerical results are generalisable – since the latter carry the risk to be parameter-specific – and that the seen transitions are indeed genuine phase transition. Hence, the results are of confirmatory and theoretical nature without expanding the biological aspects.

- Fig 4: is it true that only the first two rows of Fig 4 show the logistic function on the left and the Hill function on the right? If so, could this be made clearer in the caption? (And if not, could the caption please be reworded?)

First of all, I like to thank the reviewer for this comment, as through it, I noticed that the association of interaction function and panels was not entirely clear in Figs. 2 and 3. I have rewritten those captions to make this clearer now.

Nonetheless, in Fig. 4, itself, it is very clearly and explicitly said in the figure caption that all panels in the left column are for the logistic function and all in the right columns for the Hill function interaction (not only the top row). I do not see why this should only refer to the two top rows. Hence, I do not see how this can be said clearer.

- Please excuse this comment if I have missed it, but what are p_3 and p_4 on page 14 line 229?

I am sorry for these typos. These should read p_1 and p_2.

- Page 14: the assumption that probabilities do not depend on the cells neighbouring cell j are strong - can the implications of this assumption be discussed?

Please note that this assumption (of the MSUM approximate model studied in this section) represents a different update scheme compared to the model we used for the numerical simulations, but the overall neighborship dependencies are not different, as the joint update probabilities of i and j depend, overall, on all 6 neighbours of i and j (and i and j themselves, which, however, are fixed by the requirement that they must have opposite cell type for an update to happen). This may lead to slightly different model outputs in a non-stationary (time-dependent) state, however, in a time-invariant, ergodic stationary state, which I consider here, the outcome should be the same: For the numerical model, we update cells i and j pairwise, which depends on all 6 neighbours of both i and j at the time of this update; for the MSUM model, we update each site individually, depending only on their respective neighbours at the update time. However, because the system is ergodic, in the stationary state, sites are chosen at equal probability over time and the steady state itself is time-invariant, hence the joint update probability of both i and j depends on all 6 neighbours of both, as in the numerical model. Therefore, the two update schemes are expected to generate the same outcomes in the stationary state, which is what I study here.

I have added a comment to explain this, at the point where this approximation is introduced.

Minor (mainly typographical and admittedly petty, with apologies, especially if these comments come across as terse) comments:

- Fig 1. is slightly misleading given the model described in the paper has a square lattice, rather than a hexagonal lattice.

Fig. 1, bottom panels, are cartoonish illustrations of the real world cell arrangements. Real-world cell arrangements are more similar to hexagonal arrangements (though not exactly), this is why it is shown as such here. Nonetheless, in the model we study a square-lattice arrangement since this allows a systematic analysis and due to the universality of such lattice models we expect that the qualitative features are sufficiently captured by this. There are limitations to the square arrangement of my model when describing the real-world situations, which are discussed in the Discussion section, in particular with regards to the predicted checkerboard patterns. I have now added also a comment regarding hexagonal cell arrangements (triangular lattices) in a footnote in the Discussion section.

- Fig 1. Caption first sentence, I don't think the word `respectively' is needed? Consistency: bottom row: or (Top Row)

This has been corrected.

- Order parameter figures: The captions state that \phi is plotted with a bold line, but I believe both lines are bolded, I would suggest that the 'label' for the \phi curve be 'solid curve'.

This has been corrected.

- Page 2, line 35: what does 'more or less' mean? Is it needed?

It means that there could be a series of states which are not committed to differentiation but some are closer to commit to differentiation ('more' primed) and others further away from commitment ('less' primed). I believe that in this context, the formulation makes sense.

- Page 2, line 50: please remove the word 'are'.

I believe this word is needed here as the verb of the sentence.

- Page 3, line 72: comma not needed.

This has been corrected.

- Page 6, line 83: probability misspelt (currently spelt 'probabilitiy' - I am only stating this to help the author locate the misspelling).

This has been corrected.

- Page 7, line 140: the phrase ‘straight to’ is misleading, consider exchanging it for ‘rapidly to’.

Thanks for this suggestion. I have edited this as suggested by the reviewer.

- Page 12 after equation 11, Gillespie misspelt (currently spelt `Gillspie').

This has been corrected.

- Page 16, line 344: is the phrase ‘on the other hand’ warranted at the start of this sentence? The previous sentence has the same requirement that cell fate be reversible?

Thanks for this suggestion. I have removed ‘on the other hand’ and added an ‘also’ regarding the reversible fate in the following line.

- The concluding paragraph/sentence of the paper is long and difficult to follow, I would consider re-writing.

I have slightly rewritten this paragraph. In particular, I have split the long sentence in two.
