# Supplementary material for: Emergent order in epithelial sheets by interplay of cell divisions and cell fate regulation
Source: PLoS Comput Biol. 2024 Oct 14;20(10):e1012465. doi: 10.1371/journal.pcbi.1012465 (PMC11501039; doi:10.1371/journal.pcbi.1012465)
Supplement: Attachment — Submitted filename: PLoS_Review_13.docx [file pcbi.1012465.s001.docx]

The article “Emergent order in epithelial sheets by interplay of cell divisions and cell fate regulation” looks at how an agent based model of cell fate choice may be used to better understand emergent order of cell-fate selection, and the effects cell division of fate selection.

This work begins with a brief motivation of the problem of cell-fate selection, and then proceeds to discuss some previous attempts in a brief manner. The authors outline their simple, yet very elegant agent based model which describes cell fate selection and division. To decide how cells transition between difference cell types (in turn, how their fate is chosen), the authors propose two different limiting probability distributions, and compare the models sensitivity to them, overserving how spatial patterning arises in both cases. To very different mechanisms in cell-fate selection are described: a cells fate is determined upon division, or cells may change their fate dynamically. A qualitative analysis is provided, comparing the behaviour from the different approaches.

I have a few comments and concerns regarding the articles fit for the journal PLoS Computational Biology.

1: The article requires a more concise literature review. Ising models have been well studied in physics with many people using them to describe cell behaviour in biological tissues. Some more effort to distinguish why this is a suitable approach to understand the emergent order of cell-fate selection, and also presenting these previous approaches would make this a much stronger article in it’s current form.

2: Upon reading the article, the work should be related back to the biological system in a clearer way. There are some rather interesting results that are observed in the article, but they are not related to a biological system.

3: Within the (rather nice and appealing) simulation results shown, they are presented for only single instance results. I would like to see some analysis perform with various simulations and a statistical analysis perform, to ensure confidence in the numerical results.

4: I am concerned about this articles suitability for the journal PLoS Computational Biology. Typically, work requires a strong link to the biological system at hand. Relevant biological data needs to be incorporated. For example, if the article were to be considered for publication, I would suggest incorporating florescence imaging analysis to draw conclusion about which of the two proposed mechanisms best relate to the biology and how. Without the link, this work is much more of a theoretically valid approach.
